# Neuroligin-1 dependent phosphotyrosine signaling in excitatory synapse differentiation

**DOI:** 10.3389/fnmol.2024.1359067

**Published:** 2024-05-15

**Authors:** Zsófia Szíber, Adèle Drouet, Magali Mondin, Florian Levet, Olivier Thoumine

**Affiliations:** ^1^University of Bordeaux, CNRS, Interdisciplinary Institute for Neuroscience, IINS, UMR 5297, Bordeaux, France; ^2^University of Bordeaux, CNRS, INSERM, Bordeaux Imaging Center, BIC, UAR 3420, US 4, Bordeaux, France

**Keywords:** synapse development, adhesion molecule, neuroligin-1, phosphorylation, PSD-95 = postsynaptic density-95, Receptor Tyrosine Kinase (RTK)

## Abstract

**Introduction:**

The synaptic adhesion molecule neuroligin-1 (NLGN1) is involved in the differentiation of excitatory synapses, but the precise underlying molecular mechanisms are still debated. Here, we explored the role of NLGN1 tyrosine phosphorylation in this process, focusing on a subset of receptor tyrosine kinases (RTKs), namely FGFR1 and Trks, that were previously described to phosphorylate NLGN1 at a unique intracellular residue (Y782).

**Methods:**

We used pharmacological inhibitors and genetic manipulation of those RTKs in dissociated hippocampal neurons, followed by biochemical measurement of NLGN1 phosphorylation and immunocytochemical staining of excitatory synaptic scaffolds.

**Results:**

This study shows that: (i) the accumulation of PSD-95 at *de novo* NLGN1 clusters induced by neurexin crosslinking is reduced by FGFR and Trk inhibitors; (ii) the increase in PSD-95 puncta caused by NLGN1 over-expression is impaired by FGFR and Trk inhibitors; (iii) TrkB activation by BDNF increases NLGN1 phosphorylation; and (iv) TrkB knock-down impairs the increase of PSD-95 puncta caused by NLGN1 over-expression, an effect which is not seen with the NLGN1 Y782A mutant.

**Discussion:**

Together, our data identify TrkB as one of the major RTKs responsible for NLGN1 tyrosine phosphorylation, and reveal that TrkB activity is necessary for the synaptogenic effects of NLGN1.

## Introduction

Synapse assembly is initiated by the binding between membrane adhesion proteins expressed at the surface of neurons, leading to the recruitment of pre- and post-synaptic scaffolds and functional receptors (Missler et al., 2012). Among them, the trans-synaptic complex formed between axonal neurexins (NRXNs) and dendritic neuroligins (NLGNs) has received considerable attention over the past three decades (Craig and Kang, 2007; Südhof, 2017). Since the identification of NLGN1 as a synaptogenic molecule in a co-culture assay (Scheiffele et al., 2000), the specific role played by NLGNs at synapses remains elusive, owing to controversial results obtained by knock-down (KD), knock-out (KO), or over-expression (OE) studies in neurons. Specifically, NLGN1 OE increases both excitatory and inhibitory synapse number in a splice variant dependent fashion, while NLGN1 KD decreases excitatory synapse density with respect to neighboring neurons that express endogenous NLGN1 levels (Chih et al., 2005, 2006; Levinson et al., 2005; Ko et al., 2009; Kwon et al., 2012). Conversely, either constitutive or conditional KO of NLGN1 has no or modest effects on synaptic density and transmission (Varoqueaux et al., 2006; Mondin et al., 2011; Chanda et al., 2017).

Aside from the direct manipulation of NLGN expression level, another range of studies has focused on signaling mechanisms associated to NLGNs (Bemben et al., 2015b). In particular, several intracellular residues on NLGN1 and NLGN2 can be phosphorylated by a variety of serine/threonine and tyrosine kinases, allowing for fine tuning of NLGN trafficking and function (Bemben et al., 2013, 2015a; Giannone et al., 2013; Antonelli et al., 2014; Letellier et al., 2018; Jiang et al., 2021). Specifically, our team identified a unique tyrosine at position 782 in the intracellular gephyrin-binding motif of NLGN1, whose phosphorylation prevents gephyrin binding and instead promotes the recruitment of PSD-95 (Giannone et al., 2013). A later *in vitro* screen of the tyrosine kinases able to phosphorylate NLGN1 at this residue, followed by the use of pharmacological inhibitors highlighted the fibroblast growth factor receptor (FGFR) and the neurotrophin-activated Tropomyosin Receptor Kinase (Trk) family as major candidates (Letellier et al., 2018). Finally, the optogenetic stimulation of a photoactivatable version of the FGFR1 (Grusch et al., 2014) increased synapse density and AMPA receptor-mediated synaptic transmission in a NLGN1-dependent manner (Letellier et al., 2020). Despite these advances, the precise tyrosine kinase responsible for NLGN1 phosphorylation and the underlying activation mechanisms leading to post-synaptic differentiation remain unclear.

In this paper, we set out to specify the pathway leading to NLGN1 tyrosine phosphorylation during synapse development. Using biochemical and immunocytochemical assays in dissociated rat hippocampal neurons, associated to pharmacological or genetic manipulation of Trk members, our study points to TrkB as one of the primary receptor tyrosine kinases (RTKs) leading to NLGN1 phosphorylation and NLGN1-dependent PSD-95 recruitment at excitatory synapses.

## Results

### Inhibiting specific RTKs decreases PSD-95 recruitment to biomimetic βNRXN1-NLGN1 clusters

**Figure 1:**
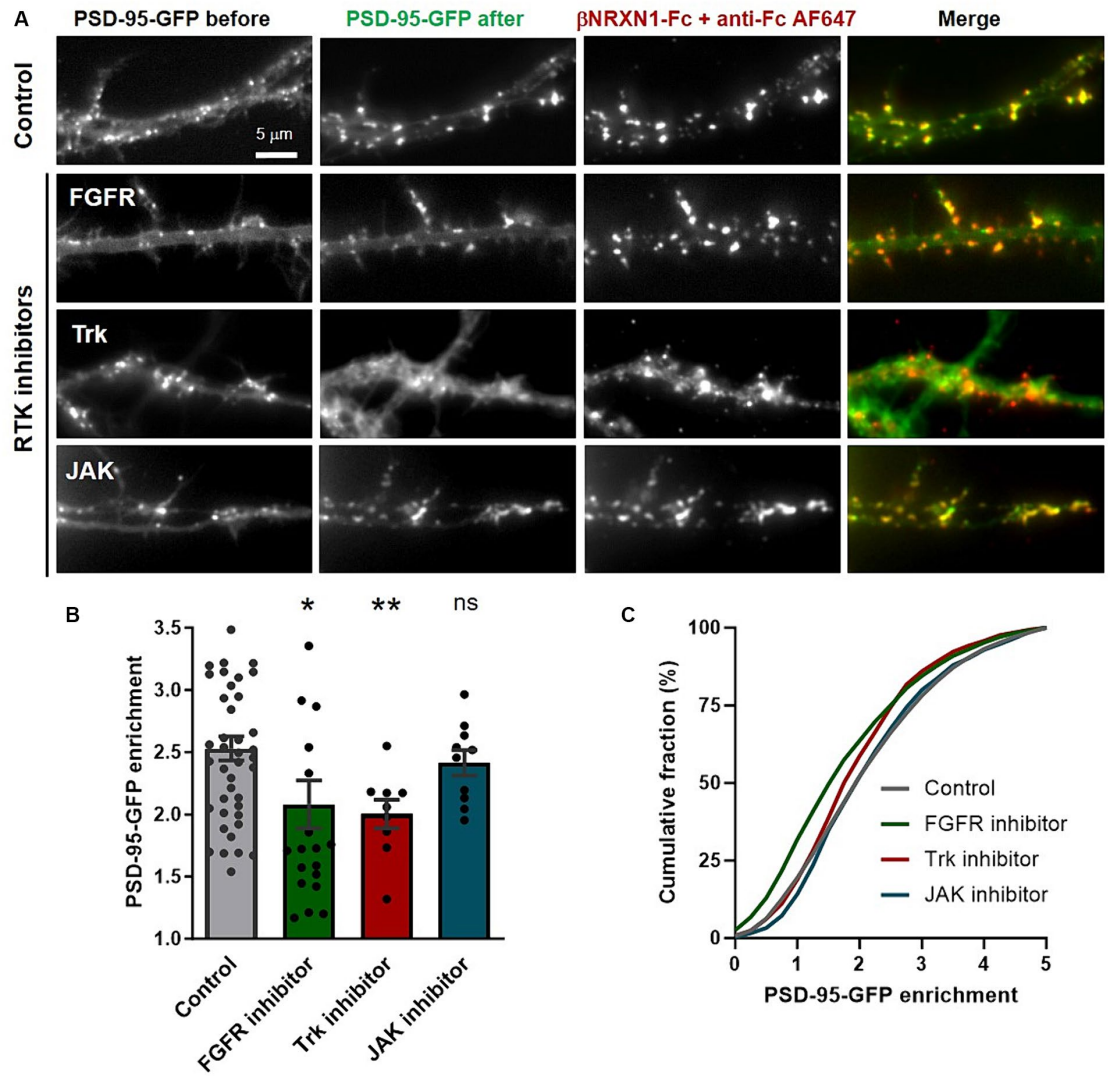
Effect of tyrosine kinase inhibitors on PSD-95 recruitment at βNRXN1-NLGN1 clusters. **(A)** Rat hippocampal neurons were co-transfected at DIV 3 with PSD-95-GFP (green), BirA^ER^, and either bAP-pDisplay or bAP-NLGN1, then observed at DIV 7. Just before the experiment, cells were pre-treated for 2 h with DMSO as vehicle (Control), FGFR inhibitor, pan Trk inhibitor, or JAK inhibitor, then incubated live for 30 min with a mix of βNRXN1-Fc and AF647-conjugated anti-human Fc (red), and directly observed live under the microscope. Note the appearance of many *de novo* PSD-95-GFP puncta which co-localize with βNRXN1-Fc clusters in the control condition, an effect which is blocked by the addition of FGFR or Trk inhibitors, but not by the JAK inhibitor. **(B)** Quantification of PSD-95-GFP enrichment in the βNRXN1-Fc clusters for the four conditions averaged per cell. The number of cells examined is given as individual dots in the columns (*n* = 9–42 from 3 independent experiments). **(C)** Corresponding cumulative distributions (the numbers of clusters analyzed were 5,694 for the control, 2,311 for the FGFR inhibitor, 1,106 for the Trk inhibitor, and 1,143 for the JAK inhibitor conditions). Unpaired data were compared by one-way ANOVA with Bonferroni correction, and FGFR and JAK inhibitors conditions were compared to the control (DMSO) by a Kruskal-Wallis test followed by a Dunn’s *post hoc* test (*p < 0.05; ns, not significant). To compare the Trk inhibitor condition to the control, a paired Mann-Whiney test was used (**p < 0.01).

We previously showed that PSD-95 recruited at NLGN1 clusters induced by βNRXN1 cross-linking was inhibited by a broad spectrum tyrosine kinase inhibitor (genistein), which led us to postulate that NLGN1 was phosphorylated (Giannone et al., 2013). Here, we used the same cluster assay in the presence of selective inhibitors of the tyrosine kinases FGFR, Trk, or JAK (Inglés-Prieto et al., 2015), that were found to phosphorylate NGLN1 by *in vitro* kinase assays (Letellier et al., 2018). Dissociated hippocampal neurons were co-transfected at DIV 3 with PSD-95-GFP and recombinant NLGN1 N-terminally tagged with the biotin acceptor peptide (bAP-NLGN1) (Howarth et al., 2005; Chamma et al., 2016), then neurons at DIV 7 were pre-treated with tyrosine kinase inhibitors or vehicle (DMSO) for 2 h, before incubation with a 2:1 mixture of βNRXN1-Fc and Cy5-conjugated anti-human Fc antibodies. This causes the specific clustering of NLGN1 and PSD-95 in a time range of 30–60 min (Mondin et al., 2011; Giannone et al., 2013). This short time window is chosen before the massive synaptogenesis period typically situated between DIV 7 and 14 in those cultures (Cottrell et al., 2000; Czöndör et al., 2012; Chanda et al., 2017), because we want to clearly distinguish *de novo* clusters of PSD-95 induced by βNRXN1 binding to NLGN1 from pre-existing synaptic PSD-95 clusters (Gerrow et al., 2006). Neurons were observed live and PSD-95-GFP enrichment (i.e., cluster versus shaft intensity level) was analyzed within βNRXN1-Fc clusters (Figure 1A). Treatments with either the FGFR inhibitor (PD166866) or the pan Trk inhibitor (GNF5837) significantly decreased the PSD-95-GFP recruitment to NLGN1 by about 30% compared to the DMSO control, while the JAK inhibitor did not have a significant effect (Figures 1B,C). Based on our previous finding that both FGFR1 and Trks are involved in the phosphorylation of endogenous NLGN1 in neurons (Letellier et al., 2018, 2020), these results indicate that NLGN1 tyrosine phosphorylation through these RTKs is implicated in the recruitment of PSD-95 to adhesive contacts formed between βNRXN1 and NLGN1.

### Inhibiting FGFR or Trks decrease the number of PSD-95 puncta induced by NLGN1 over-expression

**Figure 2:**
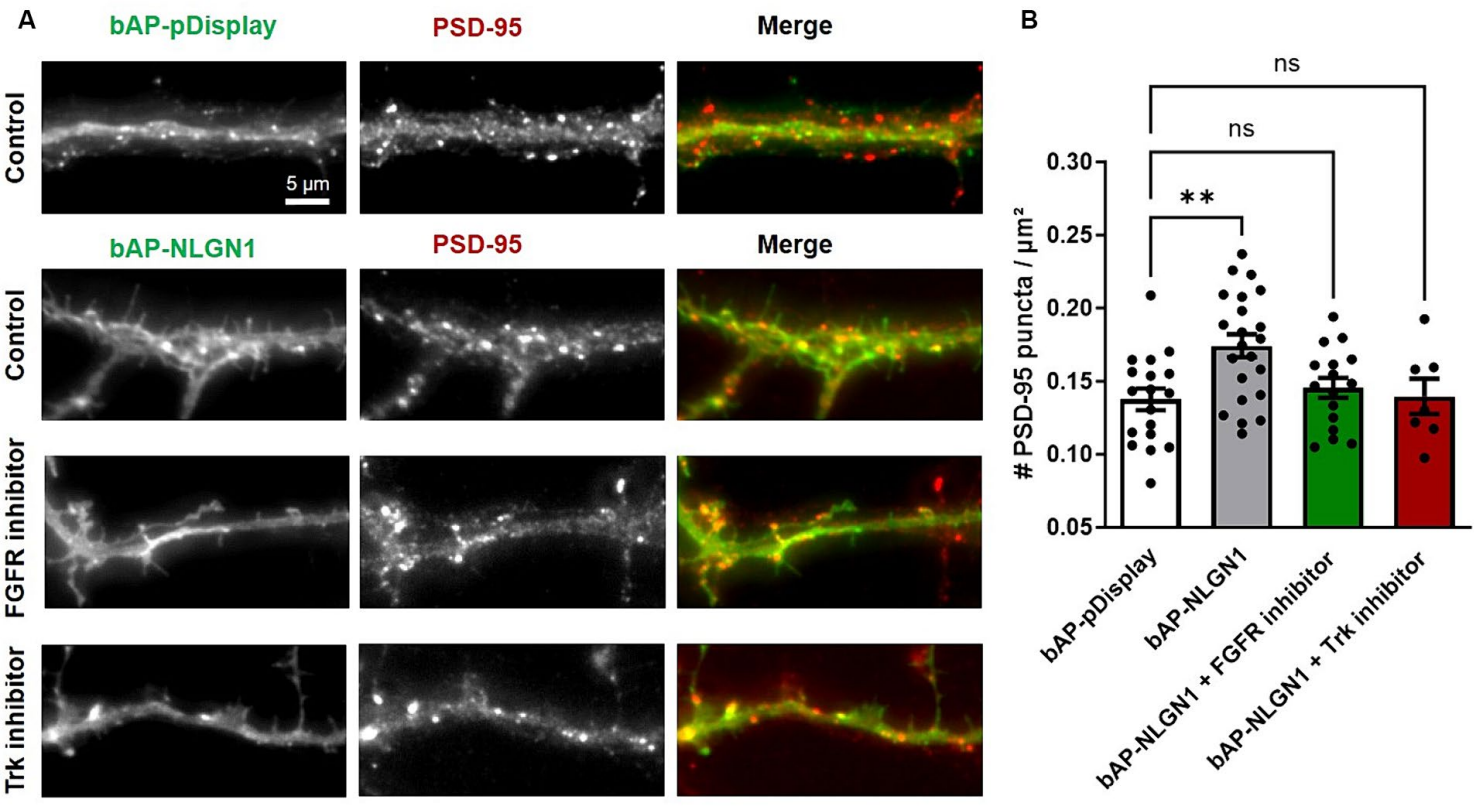
Effect of RTK inhibitors on the synaptogenic function of NLGN1. **(A)** Rat hippocampal neurons were transfected at DIV 7 with bAP-NLGN1 + BirA^ER^ or bAP-pDisplay as control, left in the incubator for 2 days in culture medium, in the presence of either DMSO vehicle (Control), FGFR inhibitor, or pan Trk inhibitor. At DIV 9, neurons were fixed and immunostained with a primary antibody to PSD-95, followed by Alexa568-conjugated secondary anti-mouse (middle panel, red in the merged images) plus Alexa647-conjugated streptavidin (left panel, green in the merged images), and observed under an epifluorescence microscope. **(B)** Quantification of the number of PSD-95 puncta per unit dendrite area in the four conditions. Data represent the average ± SEM per cell, each dot corresponding to the mean of 2–3 dendritic segments per individual neuron (*n* = 14–22 cells from 3 independent experiments). Data were compared by one-way ANOVA with Bonferroni correction, and individual conditions were compared to the control (bAP-pDisplay) by a Kruskal-Wallis test followed by a Dunn’s *post hoc* test (**p < 0.01; ns, not significant).

Over-expressing recombinant NLGN1 in neurons is well known to increase the density of excitatory synapses (Levinson et al., 2005; Chih et al., 2006; Letellier et al., 2018), and we wondered if inhibiting FGFR or Trks could also impair this synaptogenic effect of NLGN1. To this aim, we transfected neurons at DIV 7 with bAP-NLGN1 or a control membrane marker (bAP-pDisplay), together with ER-resident biotin ligase BirA^ER^ that biotinylates the bAP tag for subsequent detection by a fluorescent streptavidin conjugate (SA-AF6457). Neurons were cultured for 48 h in medium containing PD166866, GNF5837, or DMSO vehicle (control), then endogenous PSD-95 was immunostained and the number of PSD-95 puncta per unit dendrite area was determined (Figure 2A). This normalization minimizes the effect of potential variations in dendrite width. As reported previously (Giannone et al., 2013), the density of PSD-95 puncta increased significantly (by 30%) upon NLGN1 OE, and this effect was entirely reversed by both FGFR and pan-Trk inhibitors (Figure 2B), indicating that the phosphorylation of NLGN1 by those RTKs is implicated in the formation of new synapses induced by NLGN1 over-expression. Because the NRXN-NLGN complex has also been implicated in dendrite development prior to synaptogenesis (Chen et al., 2010; Constance et al., 2018), we checked for a potential effect of NLGN1 OE and RTK inhibitors on dendrite length in our conditions. There was a small, but not significant, increase in average dendrite length in neurons expressing bAP-NLGN1 as compared to neurons expressing bAP-pDisplay (Supplementary Figure S1). Moreover, treatments with either FGFR or Trk inhibitors did not reduce dendrite length in neurons expressing bAP-NLGN1. Thus, the inhibitory effects of RTKs on NLGN1-dependent synaptogenesis are not due to prior effects of NLGN1 on dendrite development.

### TrkB stimulation increases NLGN1 tyrosine phosphorylation

**Figure 3:**
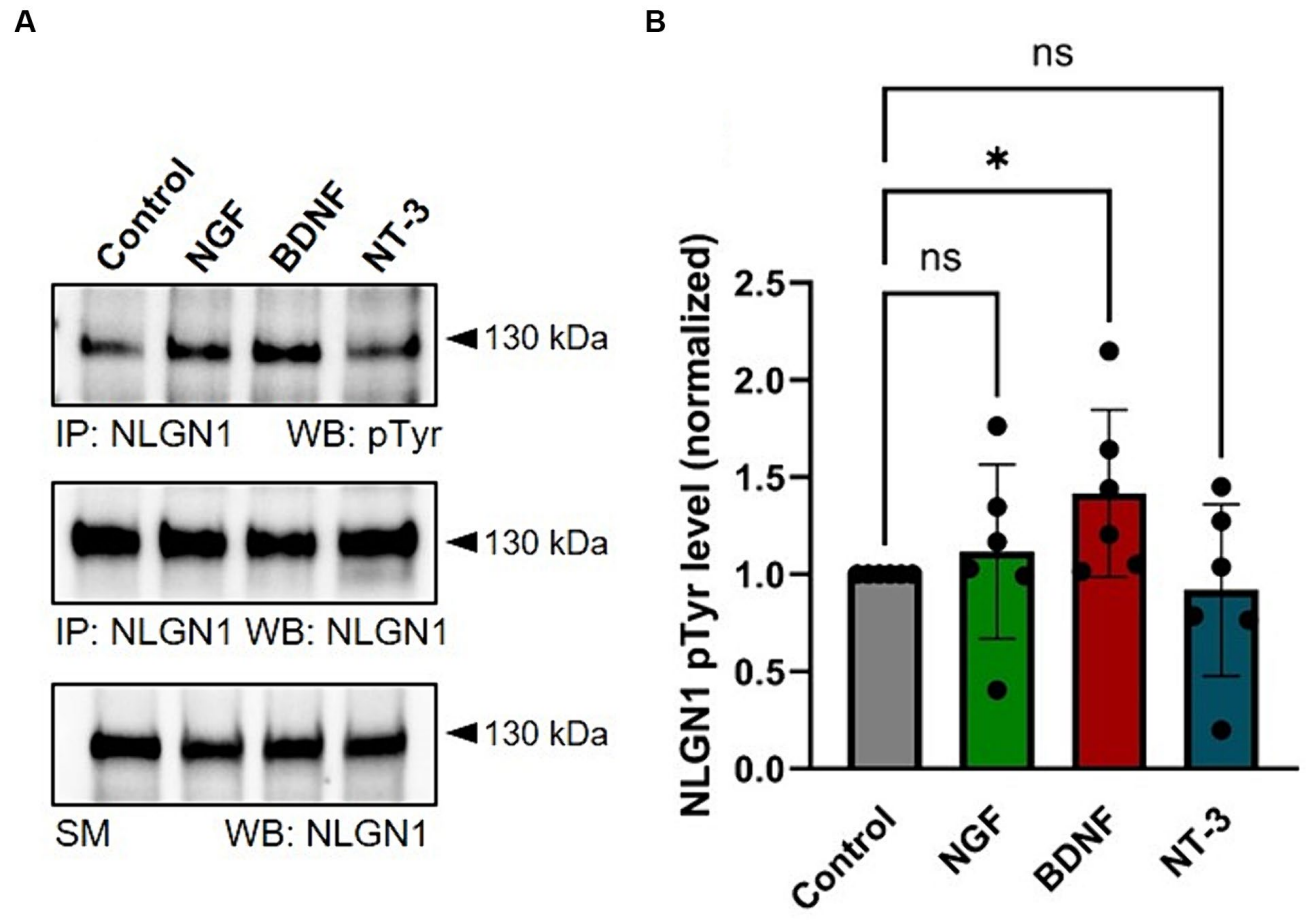
Stimulation of endogenous NLGN1 phosphorylation by Trk ligands in cultured cortical neurons. **(A)** phospho-Tyrosine (pTyr) and NLGN1 Western blots (WB) of immunoprecipitated (IP) NLGN1 upon treatment of DIV 10 primary cortical neurons with NGF, BDNF or NT-3 (lanes 2–4, respectively), as compared to untreated cultures (Control). At the bottom, the NLGN1 immunoblot is also shown for the starting material (SM). **(B)** Quantification of the NLGN1 pTyr level normalized by the NLGN1 level from the IP, in the four conditions. Mean ± SEM from 6 independent experiments. Data were compared by ANOVA followed by Kruskal-Wallis test and Dunn’s *post hoc* test (*p < 0.05; ns, not significant).

Given that the FGFR inhibitor has more modest effects on NLGN1 tyrosine phosphorylation than the pan Trk inhibitor (Letellier et al., 2018), we thereafter focused our analysis on the role of the Trk family in the regulation of NLGN1-dependent synapses. To differentiate between the various Trk members involved in NLGN1 tyrosine phosphorylation, we treated cortical neurons with ligands of TrkA, TrkB and TrkC (NGF, BDNF, and NT-3, respectively) for 20 min, followed by immunoprecipitation (IP) of endogenous NLGN1 and pTyr immunoblot (Figure 3A), as previously described (Letellier et al., 2018). We used cortical cultures instead of hippocampal cultures for those experiments because they allow us to obtain a larger number of neurons, as required for a high-yield biochemical isolation of NLGN1. Out of the three RTK ligands, BDNF was the only one to significantly increase the NLGN1 pTyr signal as compared to untreated control cultures (Figure 3B), suggesting that TrkB is a major regulator of NLGN1 tyrosine phosphorylation in neurons. Based on this result, we thus explored in more detail the function of TrkB in relation to NLGN1-dependent synapses.

### TrkB knock-down decreases the number of PSD-95 puncta induced by NLGN1 over-expression

Use of the pan-Trk pharmacological inhibitor did not allow us to discriminate which specific Trk family member was involved in the NLGN1-dependent increase in synapse formation (Letellier et al., 2018). We thus turned here to a knock-down and rescue approach, focusing on the role of TrkB in NLGN1 phosphorylation and NLGN1-dependent excitatory synapse assembly. To first check the validity of the approach, we transfected COS-7 cells with HA-NLGN1 and TrkB-RFP constructs, in the presence or not of shRNA against TrkB (sh-TrkB). Western blots performed on proteins isolated from those cell lysates showed a 70% decrease of TrkB-RFP expression by sh-TrkB, and the absence of effect on rescue TrkB-RFP (TrkB-res) (Supplementary Figures S2A,B). In parallel, the phosphotyrosine (pTyr) signal associated with NLGN1 was increased by the expression of TrkB compared to an empty vector (Supplementary Figures S2A,C). This increase was impaired by the co-expression of sh-TrkB, and reversed by the expression of rescue TrkB-RFP.

**Figure 4:**
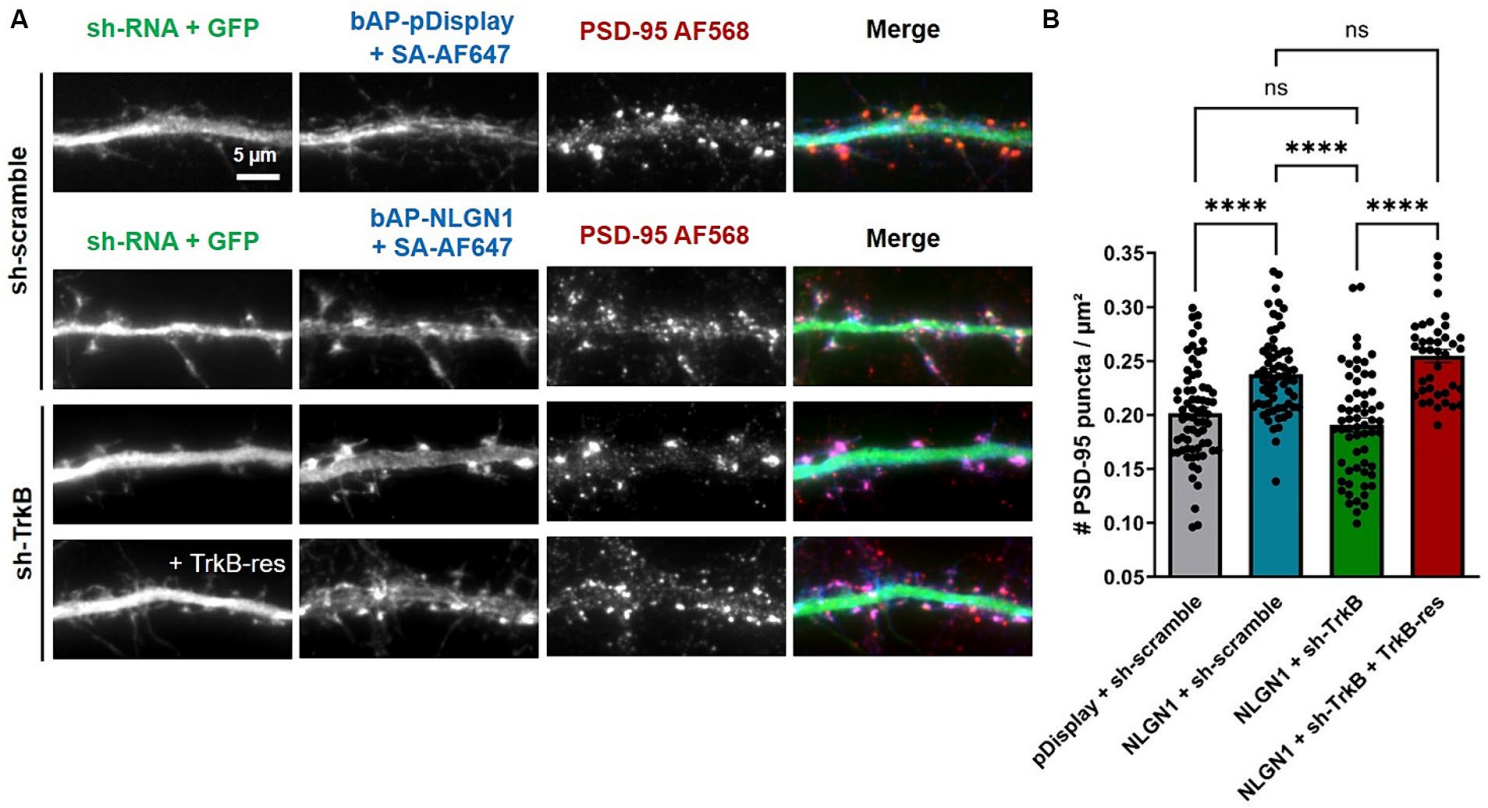
Effect of TrkB knock-down or rescue on the density of PSD-95 puncta. **(A)** Hippocampal neurons were co-transfected at DIV 7 with bAP-NLGN1 together with BirA^ER^ and TrkB constructs (sh-scramble, sh-TrkB, or sh-TrkB plus rescue TrkB-RFP). At DIV 14, neurons were fixed and dual stained for endogenous PSD-95 using primary antibody followed by AF568-conjugated secondary antibody (red in the merged images), and for biotinylated bAP-NLGN1 with streptavidin-AF647 (blue in the merged images). shRNAs have a GFP reporter shown in the first column (green in the merged images). **(B)** Graph showing the density of PSD-95 puncta per unit dendrite area in the different conditions. Two individual dendritic segments per neuron were analyzed, and data represent the average ± SEM per cell, each dot representing one neuron (*n* = 40–68 cells from 3 independent experiments). Data were analyzed by one-way ANOVA with Bonferroni correction, and conditions were compared to one another using a Kruskal-Wallis test followed by a Dunn’s *post hoc* test (****p < 0.001; ns, not significant).

Then, hippocampal neurons were co-transfected with bAP-NLGN1 (or bAP-dDisplay), BirA^ER^, and either scramble shRNA, sh-TrkB, or sh-TrkB + rescue TrkB-RFP, and neurons were stained at DIV 14 with an antibody to endogenous PSD-95 and with streptavidin-AF647 to label biotinylated proteins (Figure 4A). As shown above, the expression of bAP-NLGN1 increased the density of PSD-95 puncta by 30% as compared to neurons expressing bAP-pDisplay, both conditions in the presence of sh-scramble (Figures 4A,B). This effect of NLGN1 OE on PSD-95 was entirely blocked by the co-expression sh-TrkB, and reversed by the co-expression of rescue TrkB-RFP (Figures 4A,B). Thus, TrkB knock-down selectively impaired the synaptogenic effect of NLGN1. To prove that this effect was mediated by NLGN1 phosphorylation, we expressed a NLGN1 mutant bearing an alanine mutation of the unique tyrosine residue in the NLGN1 intracellular tail, i.e., NLGN1-Y782A (Giannone et al., 2013; Letellier et al., 2018). Because it is unable to bind gephyrin and instead binds well PSD-95, this NLGN1-Y782A mutant is thought to mimic phosphorylated NGLN1 (Giannone et al., 2013). As expected, the density of PSD-95 puncta in neurons expressing bAP-NLGN1-Y782A was as high as in neurons expressing bAP-NLGN1-WT, and not decreased by the co-expression of sh-TrkB (Supplementary Figures S3A,B). Thus, the increase in synapse formation mediated by NLGN1 involves the phosphorylation of the Y782 residue by TrkB.

### TrkB knock-down impairs the increase in spine volume induced by NLGN1 over-expression

**Figure 5:**
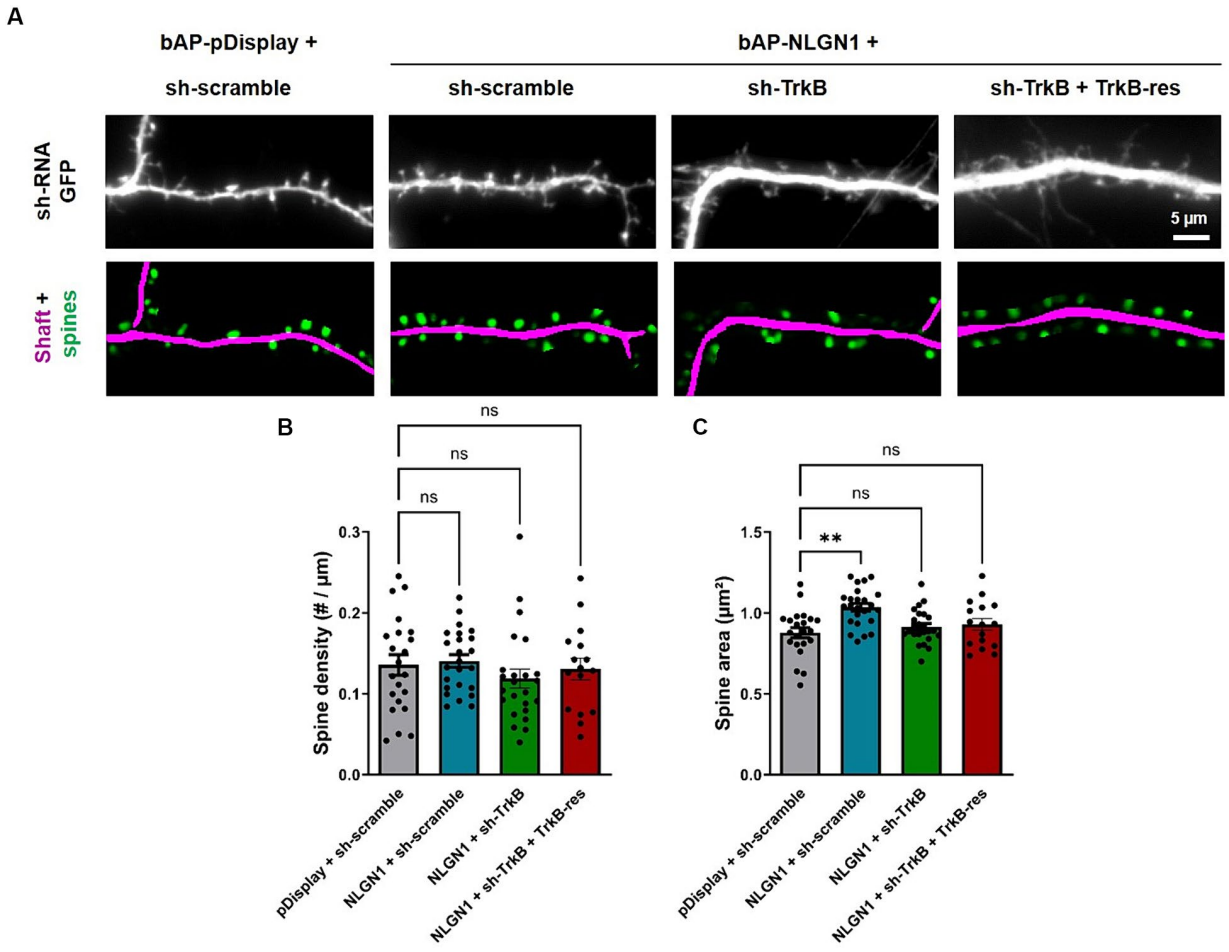
Effect of TrkB knock-down and rescue on dendritic spine density and morphology. **(A)** Hippocampal neurons were co-transfected at DIV 7 with bAP-NLGN1 together with BirA^ER^ and TrkB constructs (sh-scramble, sh-TrkB, or sh-TrkB plus rescue TrkB-RFP). At DIV 14, neurons were fixed. shRNAs have a GFP reporter shown in the top panels. The GFP images were analyzed using a deep-learning based program that segments the dendrite shaft (magenta) and the spines (green) in the corresponding bottom panels. **(B,C)** These data were used to compute the density of spines per unit dendrite length and their projected area, respectively, in all conditions. Data represent the average ± SEM per cell, each dot representing one neuron (*n* = 16–24 cells from 3 independent experiments). Data were analyzed by one-way ANOVA with Bonferroni correction, and conditions were compared to one another using a Kruskal-Wallis test followed by a Dunn’s *post hoc* test (**p < 0.001; ns, not significant).

In addition, we computed the surface density and projected area of dendritic spines by analyzing the same images taken from DIV14 neurons (Figure 5A), using a recently released program based on deep learning (Fernholz et al., 2024). Surprisingly, the linear density of spines was not altered by NLGN1 OE as compared to the pDisplay condition, and not affected either by the manipulation of TrkB levels (Figure 5B). In contrast, NLGN1 OE increased the projected area of spines (Figure 5C), an effect that may be mediated by the interaction between NLGN1 and the WAVE complex (Chen et al., 2014; Xing et al., 2018), that in turn regulates the assembly of a branched actin network in dendritic spines (Chazeau et al., 2014). Interestingly, this increase in spine volume caused by NLGN1 OE was reversed by the co-expression of sh-TrkB (Figure 5C). These data combined to the increase in PSD-95 puncta induced by NLGN1 OE suggest the formation of dendritic spines containing several post-synaptic density modules (Hruska et al., 2018).

## Discussion

By building on our previous findings that NLGN1 tyrosine phosphorylation is an important event in the differentiation of excitatory synapses (Giannone et al., 2013; Letellier et al., 2018, 2020), we explored here in detail the role of several RTKs in their ability to modulate the synaptogenic function of NLGN1. Together, our data demonstrate that both the FGFR1 and TrkB are critical in the pathway by which NLGN1 participates to excitatory synapse assembly.

Since our early report that NLGN1 is an adhesion molecule “activated” by βNRXN1 (Giannone et al., 2013), we wondered how the phosphorylation process is coupled to NRXN-NLGN1 adhesion. One mechanism can be that axonal NRXNs binds specific RTKs expressed in the dendritic membrane via extracellular interactions, bringing them to the proximity of NLGN1. Although we were unable here to specifically detect endogenous FGFR1 or TrkB in neurons due to the lack of antibodies suitable for immunofluorescence, a previous proteomics study revealed the presence of TrkB in the excitatory synaptic cleft (Loh et al., 2016). Moreover, a combination of affinity chromatography using βNRXN1-Fc as a bait followed by mass spectrometry revealed the presence of low levels of TrkB (Savas et al., 2015), indicating that TrkB might interact with βNRXN1. The related question is how RTKs would get specifically activated at the synapse? Most RTKs such as Trks and FGFRs are known to dimerize in response to extracellular ligand binding. Dimerization brings the two intracellular kinase domains in proximity, leading to cross-tyrosine phosphorylation and kinase activation (Chang et al., 2014; Grusch et al., 2014). Can the association of FGFR1 or TrkB to the NRXN-NLGN1 complex trigger such an activation? An interesting mechanism would be that NRXN1 binding to NLGN1 triggers an allosteric modification of the NLGN1 extracellular domain, that would expose binding sites for RTKs, but the crystal structure of NRXN/NLGN complexes do not reveal major conformational modifications of NRXN-occupied versus free NLGN1 (Araç et al., 2007; Fabrichny et al., 2007). Alternatively, does RTK activation rely on the specific release of ligands at excitatory synapses (Harward et al., 2016)? Our results show that TrkB activation with BDNF supplemented in the culture medium can enhance endogenous NLGN1 tyrosine phosphorylation. Interestingly, the knock-down of MDGA2, an endogenous competitor of NRXN-NLGN binding, also causes an increase of NLGN1 phosphotyrosine level (Toledo et al., 2022). Thus, perhaps the two mechanisms co-exist, i.e., recruitment of TrkB at excitatory synapses through the binding to NRXN-NLGN1 adhesions, and local activation by BDNF.

The downstream consequences of NLGN1 tyrosine phosphorylation by RTKs include the recruitment of PSD-95, as shown by the impairment of PSD-95 accumulation at βNRXN1-NLGN1 clusters by the pharmacological inhibition of either FGFR1 or Trks. Moreover, these treatments as well as the genetic invalidation of TrkB blocked the increase in PSD-95 positive synapses induced by NLGN1 over-expression. Despite the fact that these RTKs likely have other downstream targets than NLGN1 in hippocampal neurons and can influence processes such as neurite outgrowth and filopodia formation (Chang et al., 2014), several observations suggest that the effects of FGFR1 and Trks on NLGN1-dependent synaptogenesis are specifically linked to NLGN1 tyrosine phosphorylation: (i) the formation of functionally active synapses upon activation of a light-gated FGFR1 in CA1 pyramidal neurons is not seen in organotypic slices from NLGN1 KO mice (Letellier et al., 2020); (ii) the pan Trk inhibitor prevents the increase of AMPA receptor-mediated EPSCs caused by NLGN1 OE in CA1 neurons, but not that caused by the NLGN1-Y782A mutant (Letellier et al., 2018); and (iii) the increase in the density of PSD-5 puncta caused by NLGN1-Y782A OE seen here is resistant to sh-TrkB, indicating that the Y782 residue is critical for the regulation of NLGN1 phosphorylation through TrkB.

Although we still do not fully understand the mechanism, it seems that the phosphorylation of the unique intracellular tyrosine in NLGN1 which is located in the gephyrin binding motif, inhibits gephyrin binding and instead promotes the binding to PSD-95 (Giannone et al., 2013). Interestingly, even though TrkB is not present in the inhibitory synapse proteome (Loh et al., 2016; Uezu et al., 2016), the loss of TrkB reduces gephyrin localization at inhibitory synapses but does not influence the localization of NLGN2 (Chen et al., 2011). Although the conserved gephyrin binding motif in NLGN2 also contains a tyrosine (Y770) (Poulopoulos et al., 2009), our previous experiments showed that immunoprecipitated NLGN2 is not tyrosine phosphorylated in cultured neurons, while NLGN3 is strongly phosphorylated (Letellier et al., 2018). Thus, those effects of TrkB on gephyrin localization at inhibitory synapses are unlikely to be mediated by NLGN2 phosphorylation, but potentially more by NLGN3 which is present at both excitatory and inhibitory synapses (Budreck and Scheiffele, 2007), and can form heterodimers with NLGN1 (Poulopoulos et al., 2012). Note also that the process of NLGN tyrosine phosphorylation by Trks is independent of the direct role of TrkC as a synaptic organizer, which does not require catalytic activity but is mediated instead by high-affinity trans-synaptic binding to the axonal protein tyrosine phosphatase receptor PTPσ (Takahashi et al., 2011).

Coming back to NLGN1, we know that its tyrosine phosphorylation level is important for the recruitment of AMPA receptors (Letellier et al., 2018), that are most likely captured through surface diffusion by PSD-95 scaffolds connected to NRXN-NLGN1 adhesions (Mondin et al., 2011; Czöndör et al., 2013). As a consequence of the high basal level of AMPARs at synapses caused by NLGN1 tyrosine phosphorylation, LTP is reduced (Letellier et al., 2020). If TrkB is the RTK responsible for NLGN1 tyrosine phosphorylation, how can this effect be reconciled with the impairment of LTP at CA3-CA1 synapses caused by BDNF knock-out (Edelmann et al., 2014)? One possibility is that the TrkB–BDNF pathway has other targets than NLGN1 in LTP induction, e.g., PKCα (Colgan et al., 2018), or perhaps that TrkB activation is triggered aside from BDNF binding, e.g., by binding to the NRXN-NLGN complex. In fact, the use of a photoactivatable RTK (the optoFGFR1) that bypassed endogenous BDNF–TrkB signaling was able to impair LTP by directly enhancing NLGN1 phosphorylation (Letellier et al., 2020).

As a conclusion, this report reveals an important role of NLGN1 phospho-signaling by RTKs in the complex mechanisms that participate to synapse maturation. It also points to specific RTKs as potential pharmacological targets to modulate the synaptic balance in the brain, for example in the context of neurodevelopmental disorders such as autism (Bourgeron, 2015), which in some individuals has been associated to genetic mutations in NLGN1 (Nakanishi et al., 2017).

## Materials and methods

### DNA constructs

GFP-βNRXN1 (Neupert et al., 2015) was a gift from M. Missler (Münster University). Plasmids for mouse HA-NLGN1 harboring both A and B splice inserts and mouse βNRXN1 without splice site 4 (SS4-) C-terminally fused to human Fc (Dean et al., 2003) were gifts from P. Scheiffele (Biozentrum, Basel). Mouse NLGN1 containing both A and B splice inserts and N-terminally fused to the biotin acceptor peptide (bAP-NLGN1), as well as endoplasmic reticulum (ER) resident biotin ligase (BirA^ER^) (Howarth et al., 2005) were gifts from A. Ting (Stanford University, CA). PSD-95-GFP (Kuriu et al., 2006) was a gift from S. Okabe (Tokyo University, Japan). shRNA to TrkB and corresponding scramble shRNA (both containing a GFP reporter), wild type rat TrkB-RFP, and shRNA-resistant (rescue) TrkB-RFP (Jeanneteau et al., 2008) were gifts from F. Jeanneteau (Montpellier, France).

### Production of the βNRXN1-Fc protein

The protocol for the production and purification of the βNRXN1-Fc protein was previously described (Mondin et al., 2013). Briefly, the conditioned medium from a stable hygromycin-resistant HEK cell line producing βNRXN1-Fc was collected, and recombinant βNRXN1-Fc was purified on a protein G column (HiTrap Protein G HP, GE Healthcare) to a concentration of 1.0 mg.mL^−1^ after elution with glycine in tubes containing Tris buffer. The protein was stored in small aliquots at −80°C and thawed right before use.

### Hippocampal neuronal cultures

Hippocampal cultures were prepared from E18 Sprague–Dawley rat embryos obtained from gestant females purchased weekly (Janvier Labs, Saint-Berthevin, France), as described in published protocols with small modifications (Kaech and Banker, 2006; Mondin et al., 2013). Animals were handled and killed according to European ethical rules, with an authorization from the local Bordeaux Ethics committee (EC 50). Hippocampi were dissected out from whole brains under a binocular (Nikon) placed in a laminar flow hood, within a 60-mm Petri dish filled with 5 mL Hibernate medium (made of 500 mL Minimal Essential Medium - MEM, 1 g 3-(N-morpholino)propanesulfonic acid (MOPS) MOPS, 3.9 mL Glucose 45%, 5 mL Na Pyruvate, pH = 7.3). After dissection, hippocampi from 8 to 12 embryos were incubated in 5 mL 0.05% trypsin–EDTA, 10 mM HEPES at 37°C for 20 min, then cells were aspirated up and down for 20 times in a flame-polished Pasteur pipet pre-coated with horse serum. Cell concentration was determined using a Malassez cell counting chamber. Neurons were resuspended in MEM supplemented with 10% horse serum (MEM-HS) and plated at a density of 3,000 cells.cm^−2^ on 18-mm coverslips previously coated for 2 h with 1 mg.mL^−1^ polylysine in borate buffer (pH = 8.3). Three hours after plating, coverslips were flipped onto 60-mm dishes containing a glial cell layer in Neurobasal medium supplemented with 2 mM L-glutamine and 1x NeuroCult SM1 Neuronal supplement (STEMCELL Technologies) and cultured for 2 weeks at 37°C and 5% CO_2_. Astrocytes were prepared from the same embryos, plated between 20,000 and 40,000 cells per 60-mm dish and cultured in MEM (Fisher Scientific) containing 4.5 g.L^−1^ glucose, 2 mM L-glutamine, and 10% horse serum (Invitrogen) for up to 14 days before being used as feeder layers for neurons.

### Hippocampal neuron transfection

Neurons were transfected using a lipofection protocol (Effecten, Qiagen). The following combinations and concentrations of plasmids were used: (i) For the βNRXN1-Fc cluster assay in the presence of RTK inhibitors (Figure 1): 1 μg bAP-NLGN1: 1 μg PSD-95-GFP and 0.5 ug BirA^ER^ (transfection at DIV 3, observation at DIV 7); (ii) for the synapse induction assay in the presence of RTK inhibitors (Figure 2): 1 μg bAP-pDisplay or bAP-NLGN1: 0.5 μg BirA^ER^ (transfection at DIV 7, observation at DIV 9–10); for the synapse induction assay upon genetic manipulation of TrkB (Figures 4, 5): 1 μg bAP-pDisplay or bAP-NLGN1; 0.5 μg BirA^ER^; 1 μg sh-TrkB (or sh-scramble), or 1 μg sh-TrkB +1 μg rescue-TrkB (transfection at DIV 7, observation at DIV 14–15). The culture medium contains free biotin in sufficient amounts so that BirA^ER^ is able to biotinylate the bAP tag on pDisplay or NLGN1.

### βNRXN1-Fc cluster assay

DIV 7 neurons co-transfected with bAP-NLGN1 and PSD-95-GFP were pre-treated for 2 h with 0.5 μM tyrosine kinase inhibitors (FGFR inhibitor PD166866, Sigma-Aldrich PZ0114), pan-Trk inhibitor (GNF5837, Tocris 4559), pan-JAK inhibitor I (Calbiochem 420099) or vehicle (DMSO, concentration 1:2,000). Then neurons were incubated live for 30 min at 37°C with a mix of 10 μL βNRXN1-Fc (at 1 mg/mL) and 5 μL Alexa647-conjugated goat anti-human Fc (Jackson Immunoresearch 1 mg/mL) diluted in 100 μL of Tyrode solution (15 mM D-glucose, 108 mM NaCl, 5 mM KCl, 2 mM MgCl_2_, 2 mM CaCl_2_ and 25 mM HEPES, pH 7.4) supplemented with 1% immunoglobulin-free BSA (Sigma-Aldrich A7030). To save protein, coverslips were turned upside down onto the drop placed on a piece of Parafilm. After incubation, neurons were rinsed in warm Tyrode and mounted in an Inox Chamber (Life Imaging Services, Basel) under a TiE Nikon microscope thermostated at 37°C.

### Immunocytochemistry and image acquisition

Cultures were fixed for 10 min in warm 4% paraformaldehyde-4% sucrose solution and permeabilized for 5 min with 0.1% Triton X-100 in PBS. Non-specific binding was blocked with PBS containing 1% Bovine Serum Albumin (BSA Sigma-Aldrich A7030). In Figures 2, 4, neurons expressing biotinylated bAP-pDisplay or bAP-NLGN1 were stained with streptavidin-AF647 (Thermo Fisher Scientific S21374, 2 mg/mL, 1:800) and endogenous PSD-95 was immunostained with a mouse monoclonal antibody (Thermo Fisher Scientific clone 7E3-1B8, 1:400), followed by Alexa568-conjugated goat anti-mouse antibody (Thermo Fisher Scientific A11031, 2 mg/mL 1:800). In Figures 4, 5, she GFP reporter of the shRNAs was not amplified. Coverslips were mounted in Mowiol (Calbiochem).

All stainings were visualized on an inverted epifluorescence microscope (Nikon Eclipse TiE) equipped with a 60x/1.40 NA oil immersion objective and filter sets for EGFP/Alexa488 (excitation: FF01-472/30; dichroic: FF-495Di02; emission: FF01-525/30); TRITC/Alexa568 (excitation: FF01-543/22; dichroic: FF-562Di02; emission: FF01-593/40); and Cy5/Alexa647 (excitation: FF02-628/40; Dichroic: FF-660Di02; Emission: FF01-692/40) (SemROCK). Full field images of 1,200 × 1,200 pixels (pixel size = 11 μm) were acquired with an sCMOS camera (Photometrics PRIME 95B), using the Metamorph^®^ software (Molecular Devices).

### Image analysis of protein clusters, dendrite length, and dendritic spines

The detection of βNRXN1-Fc clusters and the quantification of the PSD-95-GFP enrichment level within those clusters (Figure 1) was carried out using a semi-automatic wavelet-based segmentation program written in Metamorph, as previously described (Racine et al., 2007; Czöndör et al., 2013; Mondin et al., 2013). Briefly, the user selects the appropriate wavelet planes and defines a threshold to correctly segment the βNRXN1-Fc clusters on the anti-Fc AF647 reference image. Puncta smaller than 10 square pixels are discarded. From this segmentation, a binary image is generated which is further used in Metamorph to automatically generate regions around those puncta. The regions are then transferred to the PSD-95-GFP images acquired in parallel, and the average intensity within those regions is measured. Finally, a threshold is applied on the overall image to highlight the dendrite contour and measure its area and intensity. The fluorescence intensity within puncta is divided by the intensity in the shaft, this ratio being defined as the enrichment factor. The same program was used to segment endogenous PSD-95 puncta for 2 dendritic segments per neuron and measure their number. This number is divided by the surface area of the dendritic segment to yield the surface density of PSD-95 puncta/μm^2^ (Figures 2, 4). The length of the 2–4 longest dendrites was determined manually in Metamorph (Supplementary Figure S1). The analysis of dendritic spines was performed using DeepD3, a deep learning-based framework that segments dendrite shafts and spines in a fully automated fashion (Fernholz et al., 2024). This program was applied to images of shRNA GFP reporters in DIV14 neurons, giving a binary image of dendrite shafts, an image of dendritic spines as a localization probability map, and a full list of the detected dendritic spines as regions of interest, whose number and projected area was determined in FIJI. The cumulated length of the corresponding binarized dendritic segments was determined in Metamorph for each individual neuron. The number of detected spines was then divided by dendrite length.

### COS-7 cell culture and transfection

COS-7 cells from the *European Collection of Authenticated Cell Cultures* (ECACC) purchased via (Sigma-Aldrich, Acc Nc 87021302) were cultured in DMEM (Eurobio) supplemented with 1% glutamax (Sigma-Aldrich, #3550–038), 1% sodium pyruvate (Sigma-Aldrich, #11360–070), 10% Fetal Bovine Serum (Eurobio). Cells were thawed from frozen vials at passage +4, and maintained up to passage 20. Cells were regularly tested negative for mycoplasma, using the MycoAlert detection kit (Lonza, #LT07-218). COS-7 cells were plated in 6-well plates at a density of 150,000 cells/well. After 5–6 h, cells were transfected using the X-tremeGENE HP DNA Transfection Reagent (Roche) with 1 µg HA-NLGN1 with or without RTKs (1 µg wild type TrkB-RFP, 4 µg shTrkB + 1 µg wild type TrkB-RFP, 4 µg shTrkB + rescue TrkB-RFP), Transfected cells were and left under a humidified 5% CO_2_ atmosphere (37°C) for 2 days before being processed for NLGN1 immunoprecipitation (IP).

### Cortical neurons for biochemistry

To obtain the high protein yields required to perform reproducible biochemistry experiments, we cultured cortical neurons. Whole cortices from E18 rat embryos were dissected out and cut into several thin pieces with a scalpel. Neurons were dissociated using the same procedure described above for hippocampal neurons, then plated at a density of 300,000–600,000 cells per well in 6-well plates coated with 1 mg.ml^−1^ polylysine and cultured for 10 days. Ligands specific for TrkA, TrkB, and TrkC, i.e., respectively NGF (50 ng/mL; Peprotech 450–01), BDNF (50 ng/mL; Peprotech 450–02), or NT-3 (50 ng/mL; Peprotech 450–03), were applied for 20 min to the cultures prior to cell lysis. Cultures were harvested and solubilized proteins were immediately processed for IP.

### NLGN1 immunoprecipitation

COS-7 cells or cortical neurons were treated with 100 μM pervanadate for 15 min before cell lysis, to preserve phosphate groups on NLGN1. Whole-cell protein extracts were obtained by solubilizing cells in lysis buffer (50 mM HEPES, pH 7.2, 10 mM EDTA, 0.1% SDS, 1% NP-40, 0.5% deoxycholate - DOC, 2 mM Na-Vanadate, 35 μM PAO, 48 mM Na-Pyrophosphate, 100 mM NaF, 30 mM phenyl-phosphate, 50 μM NH_4_-molybdate and 1 mM ZnCl_2_) containing protease Inhibitor Cocktail Set III, EDTA-Free (Calbiochem). Lysates were clarified by centrifugation at 8,000 × g for 15 min. For NLGN1 immunoprecipitations, 500–1,000 μg of total protein (estimated by Direct Detect assay, Merck Millipore), were incubated overnight with 2 μg of, rabbit anti-NLGN1 (Synaptic systems 129013), then precipitated with 20 μL protein G beads (Dynabeads Protein G, Thermo Fisher Scientific) and washed 4 times with lysis buffer. At the end of the IP, 20 μL beads were resuspended in 20 μL of 2x loading buffer, and supernatants were processed for SDS-PAGE and Western blotting.

### SDS-PAGE and immunoblotting

Proteins were loaded on 4–15% (Mini-PROTEAN TGX Precast Protein Gels, Bio-Rad) for separation (200 V, 400 mA, 40 min) and afterwards transferred to nitrocellulose membranes for semi-dry immunoblotting (7 min, BioRad). Membranes were rinsed in Tris-buffered saline Tween-20 (TBST; 28 mM Tris, 137 mM NaCl, 0.05% Tween-20, pH 7.4) and incubated with 5% non-fat dried milk for 45 min at room temperature. Membranes were incubated for 1 h at room temperature (or overnight at 4°C) in 0.5% non-fat dried milk in TBST containing the appropriate primary antibodies, as follows: mouse anti-pTyr (1:1,000, Cell Signaling 9411S and Merck 05–1050), rabbit anti-NLGN1 (1:1,000, Synaptic systems 129013), anti-GAPDH (1:10,000, GeneTex GTX627408), anti-GFP (1:5,000, Roche 11814460001), anti-HA (1:1,000, Ozyme BLE901513), anti-RFP (1:5,000, Abcam ab124754). After washing 3 times with TBST buffer, blots were incubated for 1 h at room temperature with horseradish peroxidase (HRP)–conjugated donkey anti-mouse or anti-rabbit secondary antibodies (Jackson Immunoresearch, 715–035-150 and 711–035-152, respectively, 1:5,000 dilution) for the SM or anti-mouse or anti-rabbit Easyblot (GeneTex, GTX221667-01 or GTX221666-01, respectively, 1:1,000 dilution) for the IP material. The latter was used to avoid the detection of primary antibodies from the IP. Target proteins were detected by chemiluminescence with Super signal West Dura or Super signal West Femto (Pierce) or Clarity Western ECL Substrate (Bio-Rad) on the ChemiDoc Touch system (Bio-Rad). Average intensity values were calculated using Image Lab 5.0 software (Bio-Rad). The ratio of phospho-NLGN1 divided by the total NLGN1 signal was normalized to the control samples.

## Data availability statement

The original contributions presented in the study are included in the article/Supplementary material, further inquiries can be directed to the corresponding author.

## Ethics statement

The animal study was approved by the Ethics Committee of the University of Bordeaux (EC-50). The study was conducted in accordance with the local legislation and institutional requirements.

## Author contributions

OT: Conceptualization, Data curation, Funding acquisition, Methodology, Project administration, Resources, Supervision, Writing – original draft. ZS: Conceptualization, Data curation, Formal analysis, Investigation, Methodology, Visualization, Writing – review & editing. AD: Data curation, Investigation, Methodology, Validation, Writing – review & editing. MM: Visualization, Software, Writing – review & editing. FL: Visualization, Software, Funding Acquisition, Writing – review & editing.
