# Supplementary material for: Neuroligin-1 dependent phosphotyrosine signaling in excitatory synapse differentiation
Source: Front Mol Neurosci. 2024 May 15;17:1359067. doi: 10.3389/fnmol.2024.1359067 (PMC11133670; doi:10.3389/fnmol.2024.1359067)
Supplement: Supplementary file 1 [file Data_Sheet_1.docx]

**Manuscript 1359067 Sziber et al.**

**Supplemental figure legends**

**Figure Supplemental 1. Effect of RTK inhibitors on the dendritogenic function of NLGN1**

**(A)** Rat hippocampal neurons were transfected at DIV 7 with bAP-NLGN1 + BirA^ER^ or bAP-pDisplay as control, left in the incubator for 2 days in culture medium, in the presence of either DMSO vehicle (Control), FGFR inhibitor, or pan Trk inhibitor. At DIV 9, neurons were fixed, stained with Alexa647-conjugated streptavidin, and observed under an epifluorescence microscope. **(B)** Quantification of dendrite length in the four conditions. Data represent the average ± SEM per cell, each dot corresponding to the mean of 2-4 of the longest dendrites per individual neuron (n = 14-22 cells from 3 independent experiments). Data were compared by one-way ANOVA with Bonferroni correction, and individual conditions were compared to the control (bAP-pDisplay) by a Kruskal-Wallis test followed by a Dunn's post hoc test (ns : not significant).

**Figure Supplemental 2. Validation of TrkB constructs on the phosphorylation of NLGN1 in COS-7 cells**

**(A)** Wild-type TrkB-RFP, sh-TrkB-GFP and rescue-TrkB-RFP constructs were co-expressed with HA-NLGN1 in COS-7 cells followed by NLGN1 immunoprecipitation (IP) and Western blot (WB) to pTyr, to detect tyrosine phosphorylated NLGN1. The starting material (SM) was immunoblotted to either NLGN1 (to detect variations in expression levels), GFP (to detect the reporter of the shRNA to TrkB), RFP (to detect TrkB-RFP fusion proteins), or GAPDH to detect any variations in total protein loading. Note that the GFP signal is present only when sh-TrkB is expressed, and the RFP signal is detected only when TrkB-RFP proteins are being expressed. **(B, C)** Graphs showing the RFP signal from the SM normalized by the GAPDH signal, and the pTyr signal normalized by NLGN1 level from the IP, respectively, in the various conditions.

**Figure Supplemental 3. Resistance of NLGN1-Y782A to TrkB knock-down on the density of PSD-95 puncta**

**(A)** Hippocampal neurons were co-transfected at DIV 7 with bAP-NLGN1-Y782A together with BirA^ER^ and either sh-scramble or sh-TrkB. At DIV 14, neurons were fixed and stained for endogenous PSD-95 using primary antibody followed by AF568-conjugated secondary antibody (red in the merged images), and counterstained for biotinylated bAP-NLGN1 with streptavidin-AF647 (blue in the merged images). shRNAs have a GFP reporter shown in the first column (green in the merged images). **(B)** Graph showing the density of PSD-95 puncta per unit dendrite area in the two conditions, in parallel to the bAP-NLGN1-WT data duplicated here from Fig. 4B for comparison. Data represent the average ± SEM per cell, each dot representing one neuron (n = 29-30 cells from 2 independent experiments). Data were analyzed by one-way ANOVA with Bonferroni correction, and conditions were compared to one another using a Kruskal-Wallis test followed by a Dunn's post hoc test (**** P < 0.001; *** P < 0.005; * P < 0.05; ns : not significant).
