# Supplementary figures and images for: Neuroligin-1 dependent phosphotyrosine signaling in excitatory synapse differentiation
Source: Front Mol Neurosci. 2024 May 15;17:1359067. doi: 10.3389/fnmol.2024.1359067 (PMC11133670; doi:10.3389/fnmol.2024.1359067)

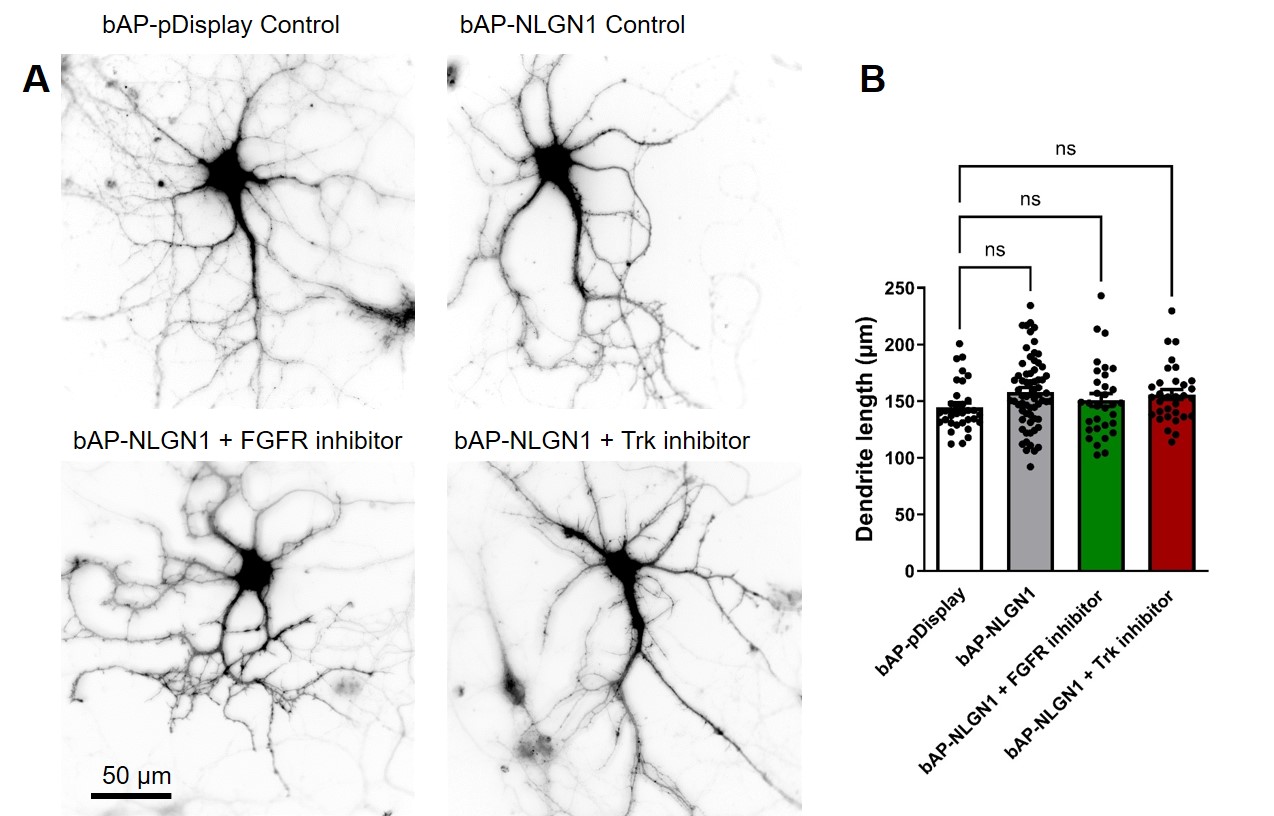

Supplement: Supplementary file 2 [file Image_1.JPEG]

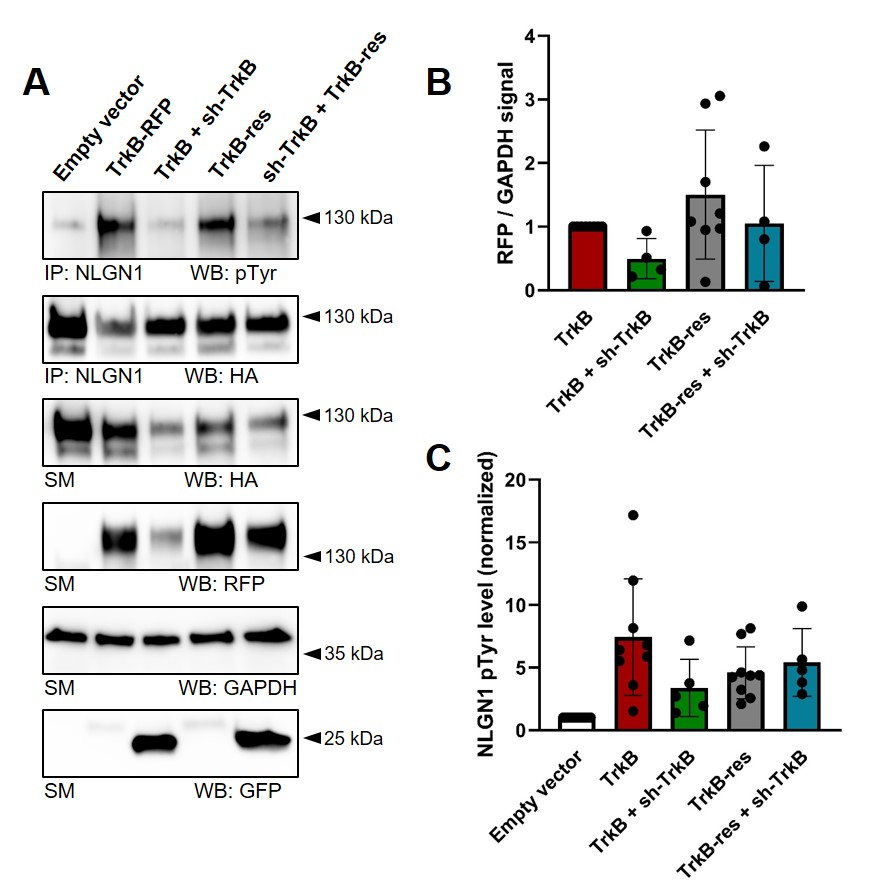

Supplement: Supplementary file 3 [file Image_2.JPEG]

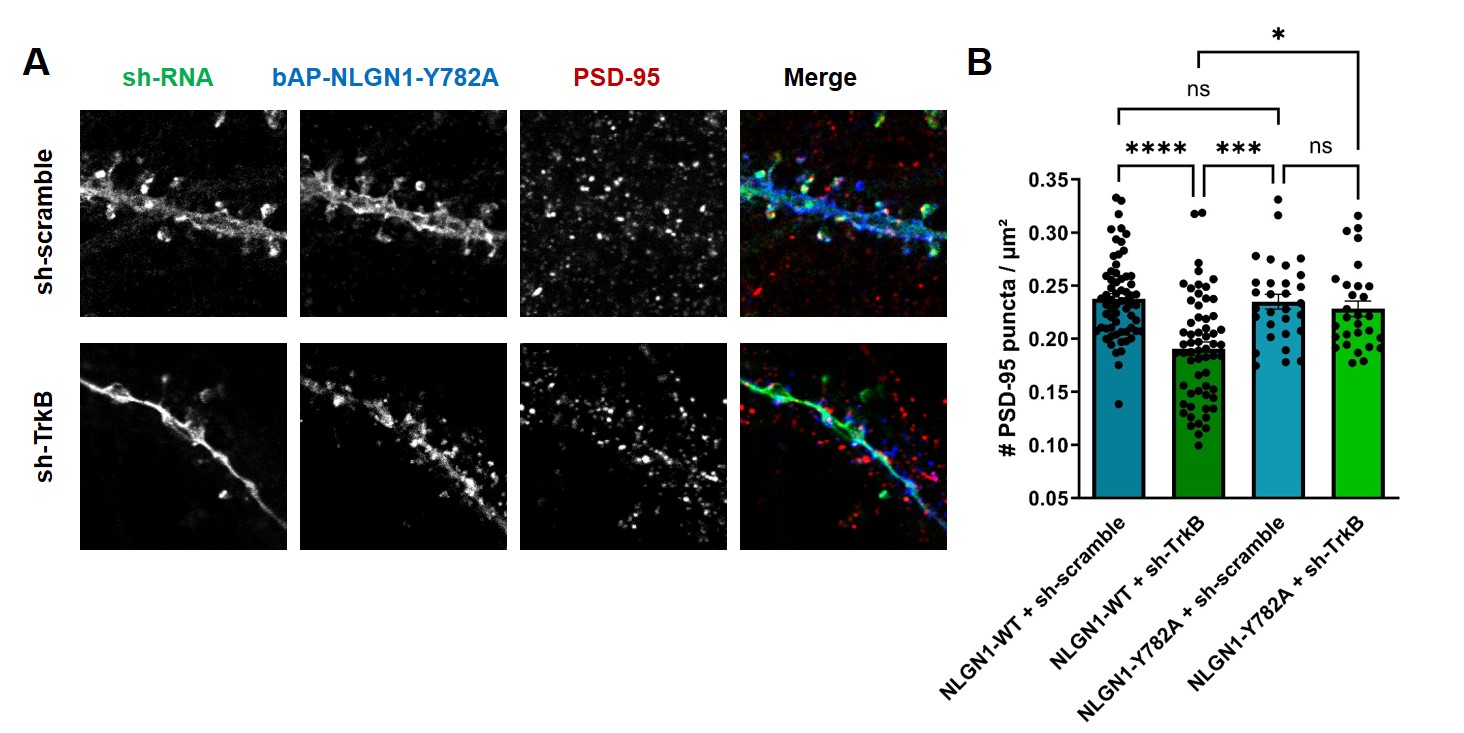

Supplement: Supplementary file 4 [file Image_3.JPEG]
